# The PathLinker app: Connect the dots in protein interaction networks

**DOI:** 10.12688/f1000research.9909.1

**Published:** 2017-01-20

**Authors:** Daniel P. Gil, Jeffrey N. Law, T. M. Murali

**Affiliations:** 1 Department of Computer Science, Virginia Tech, Blacksburg, USA; 2 Genetics, Bioinformatics, and Computational Biology, Virginia Tech, Blacksburg, USA; 3 ICTAS Center for Systems Biology of Engineered Tissues, Virginia Tech, Blacksburg, USA

**Keywords:** signaling pathways, pathway reconstruction, protein interaction networks, PathLinker, Cytoscape, k-shortest paths

## Abstract

PathLinker is a graph-theoretic algorithm for reconstructing the interactions in a signaling pathway of interest. It efficiently computes multiple short paths within a background protein interaction network from the receptors to transcription factors (TFs) in a pathway. We originally developed PathLinker to complement manual curation of signaling pathways, which is slow and painstaking. The method can be used in general to connect any set of sources to any set of targets in an interaction network. The app presented here makes the PathLinker functionality available to Cytoscape users. We present an example where we used PathLinker to compute and analyze the network of interactions connecting proteins that are perturbed by the drug lovastatin.

## Introduction

Signaling pathways are a cornerstone of systems biology. While several databases store high-quality representations of these pathways, they require time-consuming manual curation. P athL inker is an algorithm that automates the reconstruction of any human signaling pathway by connecting the receptors and transcription factors (TFs) in that pathway through a physical and regulatory interaction network ^1^. In previous work, we have demonstrated that P athL inker achieved much higher recall (while maintaining reasonable precision) than several other methods ^1^. Furthermore, it was the only method that could control the size of the reconstruction while ensuring that receptors were connected to TFs in the result. We have also experimentally validated P athL inker’s novel finding that CFTR, a transmembrane protein, facilitates the signaling from receptor tyrosine kinase Ryk to the phosphoprotein Dab2, which controls signaling to β-catenin in the Wnt pathway ^1^. These encouraging results suggest that P athL inker may serve as a powerful approach for discovering the structure of poorly studied processes and prioritizing both proteins and interactions for experimental study.

More generally, P athL inker can be useful for connecting sources to targets in protein networks, a problem that has been the focus of many studies in the past ^2– 8^. Applications have included explaining high-throughput measurements of the effects of gene knockouts ^9, 10^, discovering genomic mutations that are responsible for changes in downstream gene expression ^11, 12^, studying crosstalk between different cellular processes ^13, 14^, and linking environmental stresses through receptors to transcriptional changes ^8^.

In this paper, we describe a Cytoscape app that implements the P athL inker algorithm. We describe in detail a use case where we employ P athL inker to analyze the Environmental Protection Agency’s ToxCast data. Specifically, we compute and analyze the network of interactions connecting proteins that are perturbed in this dataset by lovastatin, a drug used to lower cholesterol. We conclude by comparing P athL inker to other path-based Cytoscape apps.

## Methods

### Implementation

**Figure 1.**
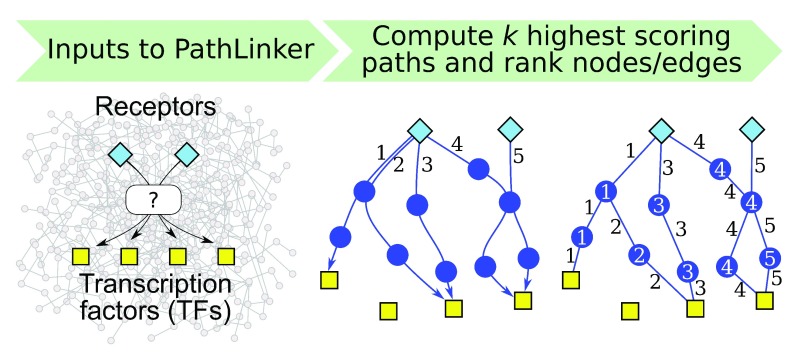
Overview of P athL inker. In this figure, P athL inker computes five paths from receptors (blue diamonds) to TFs (yellow squares) and ranks each node and edge by the index of the first path that contains it.

P athL inker requires three inputs ( Figure 1): a (directed) network *G*, a set *S* of sources, and a set *T* of targets. Each element of *S* and *T* must be a node in *G*. Each edge in *G* may have a real-valued weight. The primary algorithmic component of P athL inker is the computation of the *k* best-scoring loopless paths in the network from any source in *S* to any target in *T* ( Figure 1). By *loopless*, we mean that a path contains any node at most once. The definition of the score of a path depends on the interpretation of the edge weights, as described in “Operation.” P athL inker computes the *k*-highest scoring paths by integrating Yen’s algorithm ^15^ with the A* heuristic, which allows very efficient computation for very large *k* values, e.g., 20,000, on networks with hundreds of thousands of edges ^1^; see Table 2 below for statistics on the running time. P athL inker outputs the sub-network composed of the *k* best paths.

**Table 1.** The top 15 functional enrichment results from the ClueGO app for the Lovastatin network computed by P athL inker. The column titled “# of Genes” displays the number of genes in the P athL inker network that are annotated to that GO term/pathway. The column titled “% Associated Genes” shows the percentage of genes annotated to that term/pathway that are in the P athL inker network.

Ontology Source	Ontology Term	Corrected *p*-value	# of Genes	% Associated Genes
GO	cellular response to peptide hormone stimulus	3.17 × 10 ^–21^	22	4.1%
GO	response to insulin	6.18 × 10 ^–19^	20	4.1%
KEGG	ErbB signaling pathway	6.95 × 10 ^–17^	12	13.7%
GO	Fc receptor signaling pathway	1.62 × 10 ^–15^	17	4.0%
GO	insulin receptor signaling pathway	2.62 × 10 ^–15^	16	4.6%
KEGG	AGE-RAGE signaling pathway in diabetic complications	3.35 × 10 ^–14^	11	10.8%
KEGG	T cell receptor signaling pathway	4.62 × 10 ^–14^	11	10.5%
KEGG	Focal adhesion	5.26 × 10 ^–14^	13	6.4%
KEGG	Chronic myeloid leukemia	7.28 × 10 ^–14^	10	13.6%
KEGG	Acute myeloid leukemia	5.73 × 10 ^–13^	9	15.7%
GO	DNA-templated transcription, initiation	1.55 × 10 ^–12^	14	4.1%
KEGG	Prolactin signaling pathway	5.14 × 10 ^–12^	9	12.5%
GO	positive regulation of T cell activation	8.00 × 10 ^–12^	12	5.2%
KEGG	Chemokine signaling pathway	2.98 × 10 ^–11^	11	5.8%
KEGG	FoxO signaling pathway	3.55 × 10 ^–11^	10	7.4%

**Table 2.** Time taken by the P athL inker app using lovastatin’s and each pathway’s set of sources and targets for increasing values of *k*.

Pathway	Lovastatin	TNF *α* Pathway	TGF *β* Pathway	Wnt Pathway
# of sources	3	4	5	14
# of targets	5	44	77	14
*k*	time (sec)			
100	3.6	3.3	4.7	5.2
1,000	9.8	7.7	10.5	13.8
10,000	94.3	86.0	116.8	144.4

One of the first steps in Yen’s algorithm is to compute the shortest path from *T* to *S*. Initially, we implemented this step by running Dijkstra’s algorithm after reversing *G*. Reversing the network using the Cytoscape API proved to be time costly. Therefore, we modified our implementation of Dijkstra’s algorithm to traverse edges from target to source. Yen’s algorithm periodically requires the temporary removal of edges from the network. However, it transpires that using the Cytoscape API to delete and add edges is inefficient. Therefore, we maintain a set of "hidden edges," which our implementation of Yen’s algorithm ignores. When P athL inker completes, the app renders the computed network using the built-in hierarchical layout, if *k ≤* 200. Since this layout renders the network upside down, i.e., with source nodes at the bottom and target nodes at the top, we reflected node coordinates around the x-axis before displaying the layout.

### Operation

**Figure 2.**
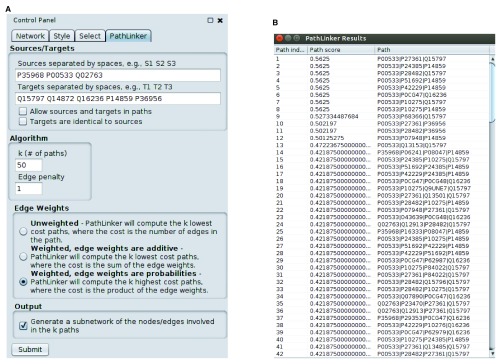
P athL inker screenshots. ( **a**) The input panel for the app. ( **b**) P athL inker lovastatin results (described in “Use Case”).

**Figure 3.**
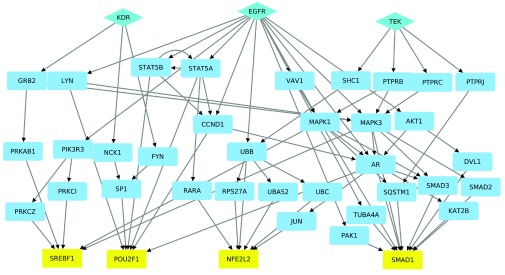
A hierarchical layout of the lovastatin network computed by P athL inker. We have mapped UniProt ids to gene names in this network.

We have implemented P athL inker in Java 7. We have tested it with Cytoscape v3.2, 3.3, and 3.4. P athL inker requires a network to be already loaded in Cytoscape. To run P athL inker on the currently selected network, the user needs to fill in the inputs and press the “Submit” button. The input panel has three sections ( Figure 2(a)):

- **Sources/Targets:** The names of the sources and the targets, separated by spaces. If there are sources or targets that are not nodes in the network, P athL inker will warn the user, identify the errant nodes, and ask the user for permission to continue with the remaining nodes. If none of the sources or none of the targets are in the network, P athL inker will exit. There are two options here:
  - **Allow sources and targets in paths:** Normally, P athL inker removes incoming edges to sources and outgoing edges from targets before computing paths. If the user selects this option, P athL inker will not remove these edges. Therefore, source and target nodes can appear as intermediate nodes in paths computed by P athL inker.
  - **Targets are identical to sources:** If the user selects this option, P athL inker will copy the sources to the targets field. This option allows the user to compute a subnetwork that connects a single set of nodes. In this case, P athL inker will allow sources and targets to appear in paths, i.e., it will behave as if the previous option is also selected. Note that since P athL inker computes loopless paths, if the user inputs only a single node and selects this option, P athL inker will not compute any paths at all.
- **Algorithm:** There are two parameters here.
  - *k* **:** the number of paths the user seeks. The default is *k* = 200. If the user inputs an invalid value (e.g., a negative number or a non-integer), P athL inker will use the default value.
  - **Edge penalty:** This value is relevant only when the network has edge weights. In the case of additive edge weights, P athL inker will penalize each path by a factor equal to the product of the number of the edges in the path and the value of this parameter. In other words, each edge in the path will increase the cost of the path by the value of this parameter. When edge weights are multiplicative, P athL inker performs the same penalization but only after transforming the weights and the edge penalty to their logarithms. The default value is one for multiplicative weights and zero for the other two cases.
- **Edge weights:** There are three options for the edge weights to be used in the algorithm:
  - **No weights:** The score of a path is the number of edges in it. P athL inker computes the *k* paths of lowest score.
  - **Edge weights are additive:** The score of a path is the sum of the weights of the edges in it. P athL inker computes the *k* paths of lowest score in this case as well.
  - **Edge weights are probabilities:** This situation arises often with protein interactions networks, since such a weight indicates the experimental reliability of an edge. P athL inker treats the edge weights as multiplicative and computes the *k* highest cost paths, where the cost of a path is the product of the edge weights. Internally, P athL inker transforms each weight to the absolute value of its logarithm to map the problem to the additive case.
- **Output:** The user can select a checkbox to generate a sub-network containing the nodes and edges in the top *k* paths. If *k ≤* 200, P athL inker will display this sub-network using the built-in hierarchical layout ( Figure 3). If *k >* 200, P athL inker will use the default layout algorithm.

When it completes, P athL inker opens a table containing the *k* paths. Each line in the table displays the rank of each path, its score, and the nodes in the path itself. The user may analyze the network computed by P athL inker using other Cytoscape apps. The next section describes a use case that further elaborates on these possibilities.

## Use Case: analysis of ToxCast data for lovastatin

The Environmental Protection Agency’s (EPA) Toxicity Forecaster (ToxCast) initiative and its extension Tox21, have screened over 9,000 chemicals (such as pesticides and pharmaceuticals) using high-throughput assays designed to test the response of many receptors, TFs, and enzymes in the presence of each chemical ^16, 17^. Here we show a use case on how to integrate P athL inker with the ToxCast data to examine possible signaling pathways by which the chemical lovastatin could affect a cell.

**Input datasets and pre-processing.** We downloaded the “ToxCast & Tox21 Summary Files” data from the ToxCast website ^18^. In these data, lovastatin perturbed three receptors (EGFR, KDR and TEK) and five TFs (MTF1, NFE2L2, POU2F1, SMAD1 and SREBF1). We used these proteins as the sources and targets, respectively, for P athL inker ( Figure 2(a)). Rather than use the default Cytoscape human network, we used the interactome used in the original P athL inker paper ^1^, which contained 12,046 nodes and 152,094 directed edges ( http://bioinformatics.cs.vt.edu/~murali/supplements/2016-sys-bio-applications-pathlinker). We preferred this network as we had used a popular Bayesian approach ^12^ to estimate edge weights so as to favor signaling interactions.

**Running P athL inker.** We used *k* = 50, no edge penalty (i.e., a penalty of 1), and the option for edge weights that indicated that they are like probabilities ( Figure 2(a)). The results appear in Figure 2(b) and Figure 3. Each row in Figure 2(b) describes a path: its index (from 1 to *k* = 50), the score of the path, and the nodes in the path, ordered from receptor to TF. Note that the score of the path is the product of the weights of the edges in it, due to the edge weight option we selected. Since P athL inker prefers high-scoring paths in this case, the paths appear in decreasing order of score. Figure 3 displays a hierarchical layout of the sub-network composed of the paths computed by P athL inker.

**Further analysis.** We mapped the node UniProt accession number names to gene names using UniProt’s ID mapping tool ( http://www.uniprot.org/uploadlists), imported the mapping results to the P athL inker network, and then changed the node labels using the *Style* tab. Finally we applied a hierarchical layout to the (lovastatin) sub-network and spread apart overlapping nodes to make the paths easier to visualize ( Figure 3). We noted that the target MTF1 did not appear in any of the top 50 paths.

**Functional Enrichment.** Since the result from P athL inker is a network in the current session of Cytoscape, it is amenable for analysis by other Cytoscape apps. As an example, we demonstrate how we applied the ClueGo app for functional enrichment ^19^ to see if the lovastatin sub-network was enriched for any Gene Ontology (GO) terms or KEGG pathways. Table 1 displays the top 15 enriched terms/pathways. Most of the paths in the P athL inker result come from the EGFR source node, so it is not surprising the ErbB signaling pathway is highly significant. We found considerable support in the literature for this pathway and other significant GO terms/pathways. Lovastatin has been shown to inhibit epidermal growth factor (EGF) and insulin-like growth factor 1 (IGF-1) ^20, 21^. Moreover, the P athL inker sub-network for lovastatin includes an interaction from EGFR to AKT1, which agrees with a study showing that lovastatin inhibits EGFR dimerization and results in the activation of AKT ^22^. Lovastatin has also been shown to inhibit the T cell receptor pathway ^23^, the Ras signaling pathway ^23^, and the Fc receptor–mediated phagocytosis by macrophages ^24^. Thus, the network computed by P athL inker for lovastatin promises to capture several possible mechanisms by which the chemical inhibits cellular pathways.

**Running time.** As we mentioned earlier in "Implementation," P athL inker is very efficient. In Table 2, we show the running time for the P athL inker app for lovastatin and for a representative set of signaling pathways. Even for *k* = 10,000, the app completed in less than 2.5 minutes for all inputs. We executed P athL inker on the same network on which we performed the lovastatin analysis.

## Comparison to related Cytoscape apps

In this section, we compare P athL inker to other Cytoscape apps that compute paths in networks. A difficulty we faced in understanding the functionality of some of these apps was that they did not precisely define their output in the documentation. Therefore, we had to take recourse to studying the source code for some of these apps in order to understand precisely the properties of the computed paths. We focus the comparison mainly on these properties and not on other features of the apps.

**PathExplorer.** ( http://apps.cytoscape.org/apps/pathexplorer) This app uses breadth first search (BFS) to compute the shortest path from a single node (that the user can select) to every other node in the network. The app can also compute the shortest path from every node in the network to a single node. Since the app uses BFS, the shortest path property is guaranteed only for unweighted networks. If there are multiple shortest paths to a node, it appears that the app will select one.

**StrongestPath.** ( http://apps.cytoscape.org/apps/strongestpath) This app computes the “strongest” paths from a group of source nodes to a group of target nodes. The authors do not provide a definition of “strongest” paths. We describe our understanding of their algorithm now. Suppose the input network is *G*. Their software takes a real-valued threshold *τ >* 0 as input; the user can manipulate a slider to select this value. The app appears to operate as follows:

1. Connect a super source *s* to each source in *G*. Connect each target to a super target *t* in *G*.
2. Use Dijkstra’s algorithm to compute the shortest path in *G* from *s* to every node in *G*.
3. Create a new network *G′ * with the same node set as *G*. For every edge ( *u*, *v*) in *G*, add the reverse of that edge ( *v*, *u*) to *G′ *.
4. Use Dijkstra’s algorithm to compute the shortest path in *G′ * from *t* to every node in *G*.
5. For every node *v* in *G*, record *d*( *v*) the sum of the length of the shortest *s*- *v* path in *G* and the length of the shortest *t*- *v* in *G′ *. Compute the corresponding *s*- *t* path *π _v_* that goes through *v*.
6. Sort all the nodes in *G* in increasing order of *d*( *v*).
7. Let *a* be the smallest value of *d*( *v*).
8. For every node *v* such that *d*( *v*) *≤ a* + *τ*, output the path *π _v_*.

In other words, for every node *v*, the app computes the shortest path that starts at some source node, goes through *v*, and ends at some target node. The number of such paths returned depends on the value of the threshold *τ* selected by the user. This app can operate on weighted and directed networks. We believe that the algorithm will compute the shortest path from any source to any target correctly. However, when *τ >* 0, it is not possible to guarantee that the algorithm will compute *all* paths from a source to a target of length *≤ a* + *τ*, since the method computes at most *n* distinct paths, where *n* is the number of nodes in the network.

**PesCa [ 25].** ( http://apps.cytoscape.org/apps/pesca30) For a single node, this app computes the shortest path from that node to every other node in the network. If the user selects multiple nodes, PesCa computes the shortest path(s) between each pair of selected nodes. A useful feature is that if there are multiple shortest paths between a pair of nodes, the app computes all of them. This app focuses on shortest paths.

**P athL inker.** Our algorithm is strikingly different in that it allows the user to compute as many ( *k*) shortest paths from sources to targets as desired. For example, if *k* = 1, P athL inker will compute the shortest path from some source to some target using Dijkstra’s algorithm on a graph with a new super source and a super target. For larger values of *k*, Yen’s algorithm (used by P athL inker) uses a dynamic program to mathematically guarantee the following property: if *π _k−_*_1_ is the ( *k −*1)st path and *π _k_* is the *k*th path, then there is no source-to-target path in the graph whose length is strictly between the lengths of *π*_*k−*1_ and *π _k_*. The other Cytoscape apps discussed here either cannot guarantee this property (e.g., StrongestPath) or do not compute less-than-optimal paths (e.g., PathExplorer and PesCa).

## Summary

We have described a new Cytoscape app that implements a mathematically rigorous, computationally-efficient, and experimentally-validated network connection algorithm called P athL inker. While we had originally developed P athL inker for reconstructing signaling pathways, the method is general enough to connect any set of sources to any set of targets in a weighted and directed network. As a specific example, we used P athL inker to compute the network of interactions connecting proteins perturbed by the drug lovastatin in the ToxCast dataset and showed how the literature supported P athL inker’s findings. The app may also be used to compute a sub-network connecting a single set of nodes. This app promises to be a useful addition to the suite of Cytoscape apps for analyzing networks.

## Data and software availability

Software available from: http://apps.cytoscape.org/apps/pathlinker

Latest source code: https://github.com/Murali-group/PathLinker-Cytoscape

Archived source code as at time of publication: 10.5281/zenodo.165162 ^26^

License: GNU General Public License version 3

The original Python implementation is available at https://github.com/Murali-group/PathLinker for users who seek to integrate P athL inker directly into their own computational pipelines or want to apply P athL inker for large values of *k*.

Datasets: We obtained the lovastatin data from the following three files in the INVITRODB_V2_SUMMARY.zip file that we downloaded ^18^:

• hitc_Matrix_151020.csv
• Chemical_Summary_151020.csv
• Assay_Summary_151020.csv

## References

[1] Ritz A Poirel CL Tegge AN: Pathways on demand: Automated reconstruction of human signaling networks. *NPJ Syst Biol Appl.* 2016;2: 16002. 10.1038/npjsba.2016.2 PMC5516854 28725467

[2] Steffen M Petti A Aach J: Automated modelling of signal transduction networks. *BMC Bioinformatics.* 2002;3(1):34. 10.1186/1471-2105-3-34 12413400 PMC137599

[3] Scott J Ideker T Karp RM: Efficient algorithms for detecting signaling pathways in protein interaction networks. *J Comput Biol.* 2006;13(2):133–144. 10.1089/cmb.2006.13.133 16597231

[4] Huang SS Fraenkel E: Integrating proteomic, transcriptional, and interactome data reveals hidden components of signaling and regulatory networks. *Sci Signal.* 2009;2(81):ra40. 10.1126/scisignal.2000350 19638617 PMC2889494

[5] Bailly-Bechet M Borgs C Braunstein A: Finding undetected protein associations in cell signaling by belief propagation. *Proc Natl Acad Sci U S A.* 2011;108(2):882–887. 10.1073/pnas.1004751108 21187432 PMC3021011

[6] Gitter A Klein-Seetharaman J Gupta A: Discovering pathways by orienting edges in protein interaction networks. *Nucleic Acids Res.* 2011;39(4):e22. 10.1093/nar/gkq1207 21109539 PMC3045580

[7] Tuncbag N Braunstein A Pagnani A: Simultaneous reconstruction of multiple signaling pathways via the prize-collecting Steiner forest problem. *J Comput Biol.* 2013;20(2):124–136. 10.1089/cmb.2012.0092 23383998 PMC3576906

[8] Gitter A Carmi M Barkai N: Linking the signaling cascades and dynamic regulatory networks controlling stress responses. *Genome Res.* 2013;23(2):365–376. 10.1101/gr.138628.112 23064748 PMC3561877

[9] Ourfali O Shlomi T Ideker T: SPINE: a framework for signaling-regulatory pathway inference from cause-effect experiments. *Bioinformatics.* 2007;23(13):i359–66. 10.1093/bioinformatics/btm170 17646318

[10] Shih YK Parthasarathy S: A single source *k*-shortest paths algorithm to infer regulatory pathways in a gene network. *Bioinformatics.* 2012;28(12):i49–i58. 10.1093/bioinformatics/bts212 22689778 PMC3371844

[11] Suthram S Beyer A Karp RM: eQED: an efficient method for interpreting eQTL associations using protein networks. *Mol Syst Biol.* 2008;4:162. 10.1038/msb.2008.4 18319721 PMC2290938

[12] Yeger-Lotem E Riva L Su LJ: Bridging high-throughput genetic and transcriptional data reveals cellular responses to alpha-synuclein toxicity. *Nat Genet.* 2009;41(3):316–323. 10.1038/ng.337 19234470 PMC2733244

[13] Yosef N Ungar L Zalckvar E: Toward accurate reconstruction of functional protein networks. *Mol Syst Biol.* 2009;5:248. 10.1038/msb.2009.3 19293828 PMC2671920

[14] Yosef N Zalckvar E Rubinstein AD: ANAT: a tool for constructing and analyzing functional protein networks. *Sci Signal.* 2011;4(196):pl1. 10.1126/scisignal.2001935 22028466

[15] Yen JY: Finding the *k* shortest loopless paths in a network. *Manage Sci.* 1971;17(11):712–716. 10.1287/mnsc.17.11.712

[16] Judson RS Houck KA Kavlock RJ: *In vitro* screening of environmental chemicals for targeted testing prioritization: the ToxCast project. *Environ Health Perspect.* 2010;118(4):485–492. 10.1289/ehp.0901392 20368123 PMC2854724

[17] Tice RR Austin CP Kavlock RJ: Improving the human hazard characterization of chemicals: a Tox21 update. *Environ Health Perspect.* 2013;121(7):756–765. 10.1289/ehp.1205784 23603828 PMC3701992

[18] USEPA: ToxCast & Tox21 Summary Files from invitrodb_v2. 2015; Data released October 2015. Reference Source.

[19] Bindea G Mlecnik B Hackl H: ClueGO: a Cytoscape plug-in to decipher functionally grouped gene ontology and pathway annotation networks. *Bioinformatics.* 2009;25(8):1091–1093. 10.1093/bioinformatics/btp101 19237447 PMC2666812

[20] Vincent TS Wülfert E Merler E: Inhibition of growth factor signaling pathways by lovastatin. *Biochem Biophys Res Commun.* 1991;180(3):1284–1289. 10.1016/S0006-291X(05)81334-8 1953779

[21] McGuire TF Xu XQ Corey SJ: Lovastatin disrupts early events in insulin signaling: a potential mechanism of lovastatin’s anti-mitogenic activity. *Biochem Biophys Res Commun.* 1994;204(1):399–406. 10.1006/bbrc.1994.2472 7524501

[22] Zhao TT Le Francois BG Goss G: Lovastatin inhibits EGFR dimerization and AKT activation in squamous cell carcinoma cells: potential regulation by targeting rho proteins. *Oncogene.* 2010;29(33):4682–4692. 10.1038/onc.2010.219 20562912

[23] Goldman F Hohl RJ Crabtree J: Lovastatin inhibits T-cell antigen receptor signaling independent of its effects on ras. *Blood.* 1996;88(12):4611–4619. 8977253

[24] Loike JD Shabtai DY Neuhut R: Statin inhibition of Fc receptor-mediated phagocytosis by macrophages is modulated by cell activation and cholesterol. *Arterioscler Thromb Vasc Biol.* 2004;24(11):2051–2056. 10.1161/01.ATV.0000143858.15909.29 15345508

[25] Scardoni G Tosadori G Pratap S: Finding the shortest path with PesCa: a tool for network reconstruction [version 2; referees: 2 approved, 2 approved with reservations]. *F1000Res.* 2015;4:484. 10.12688/f1000research.6769.2 27781081 PMC5054806

[26] Gil D Bezawada S Murali TM: The PathLinker App for Cytoscape [Data set]. *Zenodo.* 2016 Data Source

