# Supplementary material for: The PathLinker app: Connect the dots in protein interaction networks
Source: F1000Res. 2017 Jan 20;6:58. [Version 1] doi: 10.12688/f1000research.9909.1 (PMC5365231; doi:10.12688/f1000research.9909.1)
Supplement: Peer review history

# Peer review: Referee response for version 1

**DOI:** 10.5256/f1000research.10680.r20285

**Published:** 2017-03-22

**Authors:** Stefan Wuchty

**Affiliations:** 1 Department of Computer Science, Center for Computational Science, University of Miami, Coral Gables, FL, USA

The manuscript 'The PathLinker app: Connect the dots in protein interaction networks' by Gil, Law and Murali introduces a Cytoscape app that allows the user to apply their PathLinker algorithm to find potential signaling pathways from a user-defined set of sources, targets and molecular interaction data. The underlying PathLinker algorithm has been introduced in the paper in Ritz *et al.*, NPJ Syst. Biol. Appl. 2016, 2:16002, indicating that the current manuscript is an extension in that it provides a Cytoscape application. The manuscript provides a crash-course in using the PathLinker algorithm, allowing the reader to quickly get into the game determining signalling paths based on the users data. As it stands, it seems to be a popular one and will be used frequently.

 While the manuscript gives enough information to get the user going, I would add a bit more information about the specifics of the underlying algorithm. It is based on Yen's algorithm but uses the A* algorithm instead of a shortest path algorithm. While many readers are probably familiar with the latter, the A* algorithm may need an introduction to avoid that users operate a 'black box'. In particular, the A* algorithm makes at each step an assessment of the distance to a target to find an optimal path. In this regard, it would be beneficial to add more details how this assessment works and the ways in which A* was embedded in the framework of Yen's algorithm. As for the latter, also Yen's algorithm deserves more detail as it is an algorithm that users rather rarely encounter to make the user fully aware what she is doing. In particular, such considerations are important as the authors describe in the paper different weights on interactions that may be used in different ways to assess and find optimal paths.

 With that said, a bit more technical information about the 'ingredients' of the algorithms that are used to compare (with regard to the ways weighting information is used) would be helpful too. Such details would allow the reader to see where the differences to (and the advantages of) the PathLinker algorithm and apps are.

I have read this submission. I believe that I have an appropriate level of expertise to confirm that it is of an acceptable scientific standard.

# Peer review: Referee response for version 1

**DOI:** 10.5256/f1000research.10680.r20283

**Published:** 2017-03-13

**Authors:** Tamás Korcsmáros, David Fazekas

**Affiliations:** 1 Earlham Institute, Norwich, UK; 2 Eotvos Lorand University, Budapest, Hungary

The paper of Gil et al. describes a new Cytoscape App, Pathlinker, which is the Cytoscape implementation of the previously published approach by the Murali group with the same name. It is always useful for the community to implement network analyzing algorithms to Cytoscape. 

 The paper and the abstract is well written and clear. The figures were well selected.

 In order to facilitate the application of the PathLinker App, it would be useful to provide more, tutorial type comments and guidelines for new users. Given the important task PathLinker is meant to solve, many users would find it useful. Currently the Methods section contains the key steps but it does not read as a protocol or suggest alternatives for troubleshooting.

 The current version of the paper does not contain the limitations of PathLinker. When this App should not be used, for which datatypes it is not good, or cases when the user should pay attention to any bias or problem?

 The comparison with existing Apps focuses on the differences in the algorithms. As this is an App paper, it would be useful to include a comparison of the functional differences (features) between the Apps.

 If possible, maybe for a new version, it would be nice if the App allows to input the source and target node names by node selection function, instead of typing it in (or pasting it in) to the requested fields.

 Finally, a small bug in the App: When the user select the checkbox to generate a sub-network as an output, it does not generate a subnetwork within Cytoscape but a new network. The problem with this that it means the attributes of the original network will be lost. This should be fixed easily.

 I believe PathLinker will be a popular and often used App for the biomedical and systems biology communities. I think the next step to increase its impact is to make the application of it as clear and as didactical as possible.

We have read this submission. We believe that we have an appropriate level of expertise to confirm that it is of an acceptable scientific standard, however we have significant reservations, as outlined above.

# Peer review: Referee response for version 1

**DOI:** 10.5256/f1000research.10680.r19605

**Published:** 2017-02-01

**Authors:** Barry Demchak

**Affiliations:** 1 Department of Medicine, University of California, San Diego, La Jolla, CA, USA

This paper describes the PathLinker Cytoscape app, including the mathematical algorithms and a comparison to similarly-focused Cytoscape apps. It is well written and address the important problem of deducing relationships that can advance biology.

 It is very economical in its explanation of the app/algorithm, its uses and its relationship to other apps, and in several places needs more explanation. Explanations tend to weigh in favor of expert Cytoscape users, though this app would be of interest to less expert users, too, particularly those trying to relate PathLinker to biological investigation. The paper would benefit from better enabling the reader to follow a use case in Cytoscape using actual data and actual app settings.

1. In Methods | Operation, please explain how to acquire and run PathLinker.
2. In "Allow sources and targets in paths" and "Targets are identical to sources", please explain the biological implications of these settings ... it's difficult to jump from the graph implications to the biological implications.
3. In "Algorithm", why is the default chosen, and what are the biological ramifications of choosing a higher or lower *k*?
4. The output in Figure 2B seems to be a standalone window. How can the user capture the results? It's unclear how the user should be using this report in investigating relationships.
5. In "Edge penalty", please explain when a edge penalty would be used in a network and what its biological implication would be.
6. In "Input datasets and pre-processing", I attempted to download the ToxCost data and could not. The site requires a credential and does not give instructions regarding how to get the credential. Without this data, the user is hard pressed to reproduce these results and then evolve his/her own questions. The web site apparently identifies this data as freely available. Can it be included as supplementary material (as a Cytoscape session file?) to assist the user in following this paper?
7. In "Input datasets and pre-processing", I tracked down the referenced original PathLinker paper. It took a while to determine which network was being used. I downloaded it and imported it into Cytoscape. During the import, there were a number of options available, and it was unclear which options should be chosen. Can this network be included as supplementary material (as a Cytoscape session file?) to assist the user in following this paper?
8. In "Running PathLinker", can you explain the biological ramifications behind the *k*=50 and edge penalty settings?
9. In "Further Analysis", can you explain which Cytoscape tool or feature you used to spread the nodes apart? I'm thinking of the biological user that's trying to follow the paper.
10. In "Functional Enrichment", can you specify which ClueGO settings you used? This is a very valuable step, and it's hard for the user to follow without giving settings.
11. In "Running Time", how many CPU cores and how much RAM were on the test machine?
12. In the "Comparison to related Cytoscape Apps", the discussion focuses on differences in graph analysis approaches, and assumes the reader can appreciate the reasons why PathLinker gives better results. The discussion could use a little more justification, and also some grounding in the biological consequences of these differences.
13. In the Introduction, the claim "any human signaling pathway" is overbroad. I suggest claiming "human signaling pathways".

I have read this submission. I believe that I have an appropriate level of expertise to confirm that it is of an acceptable scientific standard, however I have significant reservations, as outlined above.
